# Mixed-mode resins: taking shortcut in downstream processing of raw-starch digesting α-amylases

**DOI:** 10.1038/srep15772

**Published:** 2015-10-23

**Authors:** Nikola Lončar, Marinela Šokarda Slavić, Zoran Vujčić, Nataša Božić

**Affiliations:** 1 Department of Biochemistry, Faculty of Chemistry, University of Belgrade, Studentski trg 12-16, Belgrade, Serbia; 2 Centre of Chemistry, Institute of Chemistry, Technology and Metallurgy, University of Belgrade, Studentski trg 12-16, Belgrade, Serbia; 3 Center of Excellence for Molecular Food Sciences, University of Belgrade, Belgrade, Serbia

## Abstract

*Bacillus licheniformis* 9945a α-amylase is known as a potent enzyme for raw starch hydrolysis. In this paper, a mixed mode Nuvia cPrime™ resin is examined with the aim to improve the downstream processing of raw starch digesting amylases and exploit the hydrophobic patches on their surface. This resin combines hydrophobic interactions with cation exchange groups and as such the presence of salt facilitates hydrophobic interactions while the ion-exchange groups enable proper selectivity. α-Amylase was produced using an optimized fed-batch approach in a defined media and significant overexpression of 1.2 g L^−1^ was achieved. This single step procedure enables simultaneous concentration, pigment removal as well as purification of amylase with yields of 96% directly from the fermentation broth.

In the past decades upstream processing has enabled remarkably high yields of industrially relevant proteins. This development imposed new challenges for the protein purification field and traditional purification schemes had to be abandoned. New problems arose, such as a higher viscosity of protein solutions which prevents direct loading on chromatography columns. Alternative solutions have found their way to industrial applications, such as PEG precipitation of monoclonal antibodies^1^,^2^. Thermostability of proteins that originate from thermophilic and extremophilic organisms can be exploited for heat treatment purification as host proteins are mostly denatured by this procedure^3^. Furthermore, secreted expression of recombinant proteins is favourable because the extract is free of a large variety of contaminant proteins normally present in the cell. But a drawback of this procedure is handling large volumes of liquid in terms of concentrating and desalting in order to prepare the extract for ion-exchange (IEX) chromatography. In these cases ultrafiltration is applied to concentrate the extract and a buffer exchange performed to obtain a sufficiently low ionic strength and an appropriate pH for subsequent purification steps. Nuvia™ cPrime™ is a hydrophobic cation exchange resin that contains a phenolic ring which mediates hydrophobic interactions and a carboxylic group that serves as cation-exchanger^4^,^5^. This design is promising for the initial capture steps^6^,^7^. Comparable resins are readily available^4^.

In spite of the extensive studies concerning the structure and thermal properties of *B. licheniformis* amylase and the numerous reports in the literature referring to the molecular mechanism of its irreversible thermoinactivation, little attention has been paid to its enzymological characterisation^8^. Detailed knowledge about the subsite architecture of *B. licheniformis* amylase is scarce^8^,^9^. Reports on the kinetics and mode of action of this industrially important enzyme cannot be found in the literature, especially when raw starch is used as a substrate. Enzyme preparations of high purity are required for mechanistic studies and improving downstream processing (DSP) is very beneficial.

A peculiarity of raw starch digesting enzymes is their adsorption on raw starch granules *via* a carbohydrate binding domain or by surface binding sites^10^. In a majority of cases, surface binding sites consist of exposed tyrosine and tryptophan residues on the surface of the enzyme (Fig. 1). Hydrophobic interaction chromatography is normally destructive towards the target protein and results in lower yields. However, in the case of raw starch digesting amylase (RSDA), hydrophobic interactions are a property of substrate binding and hence, high recovery is expected from a mixed mode resin. Herein, the complete workflow of overproduction of RSDA in a laboratory fermenter and proposed DSP is described.

## Results and Discussion

### Production of recombinant *RSDA* in fed-batch cultures

**Figure 2:**
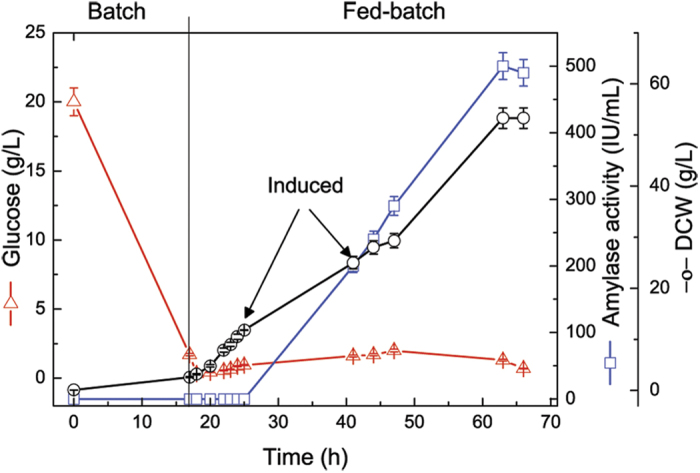
Fed-batch culture course of *E. coli* C43/pDA-amy., -○- DCW, -□- enzyme activity, -Δ- glucose. Each data point represents the mean of three independent assays (the standard errors were less than 5% of the means). The arrow indicates the point of induction.

A constant glucose supply, while providing enough oxygenation at an exponential stage of growth, enables reaching high cell densities. This approach offers a tool for increasing the yield of recombinant enzyme production. A two-stage feed strategy was applied to achieve high-cell-density in the cultivation of *E. coli* C43 (DE3) and production of recombinant α-amylase. *E.coli* C43(DE3) cells readily express genes cloned into any T7 vector and is a BL21(DE3) derivative effective in expressing toxic and membrane proteins. During the pre-induction phase, the glucose feed rate was increased exponentially according to the exponential feed method^11^, and the cell growth was controlled at a specific growth rate of 0.20 h^−1^. During a post-induction phase, a low constant feed rate was applied because applying the same exponential feed strategy during the post-induction phase might cause the accumulation of nutrients in the medium. This is usually a consequence of changes in the host cell physiology and metabolism after induction. When the dry cell weight (DCW) reached a value of 15 g L^−1^, the post-induction phase began and the glucose feed rate was kept constant at 15 mL h^−1^. When the DCW reached a value of 25 g L^−1^, the glucose feed rate was reduced to 5 mL h^−1^. The DCW of *E. coli* cells increased from 0.11 to 52.3 g L^−1^ (Fig. 2). In order to lower the extent of the metabolic burden, the optimal point for amylase induction was investigated in the previous work and was shown to be suitable at an intermediate cell density. The induction temperature is an important parameter for recombinant protein production in *E. coli*^12^,^13^. In general, the growth of recombinant *E. coli* cells at low temperatures increases the solubility of the intracellular recombinant proteins by preventing the formation of inclusion bodies. Induction at 25 °C attained the highest total amylase yield in our previous study^14^. This suggests that low culture temperature facilitates conformational quality and functionality of the protein and thereby improves the productivity of amylase.

Through this cultivation approach, the total amylase activity reached 500 U mL^−1^, which was a 2-fold higher than in fed-batch culturing of *E. coli* BL21 (DE3). The content of RSDA at the end was 1.2 g L^−1^.

### Purification of amylase on mixed mode resin

The reusability of ion-exchangers is often hampered by the efficiency of their sanitization (cleaning in place)^15^,^16^. Anion exchangers are more prone to strongly binding pigments and other difficult to remove colouring substances^17^,^18^. Cation exchangers are easier to maintain in this regard and the investigated mixed mode resin is similar in this manner. All pigments present in the fermentation broth come off the column in the flow through fraction and during the washing step with the starting buffer. This is important because broth pigments stuck to both ultrafiltration membranes in the traditional method of concentrating as well as Sepharose-based IEX resins^18^,^19^.

**Figure 3:**
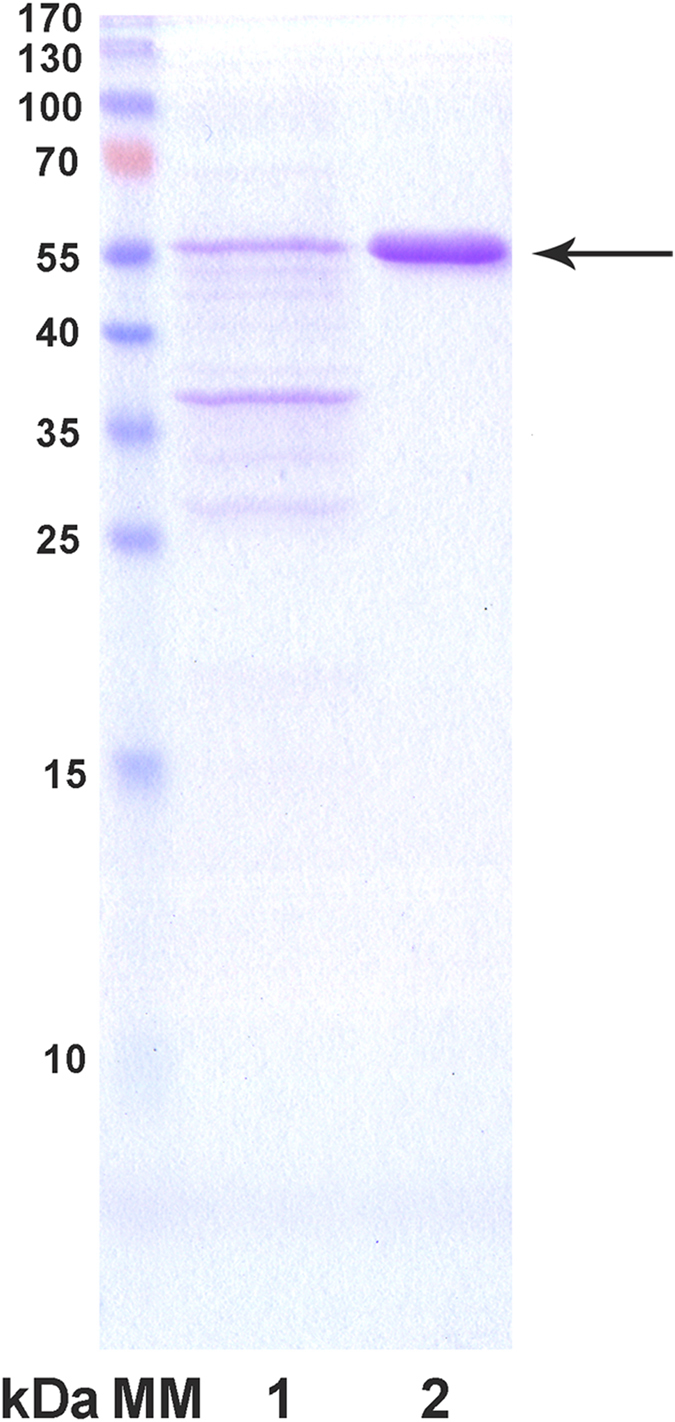
SDS PAGE analysis. Lane M: molecular markers; lane 1: cell free extract (fermentation broth); lane 2: purified amylase.

Binding of the desired enzyme to the resin is not as easy to predict compared to traditional IEX and several pilot experiments are necessary. Surface response methodology was used to optimize the binding and eluting conditions of RSDA (Supplementary Fig. S1). In the chosen purification strategy, flow through fractions tested for amylase activity showed a total of 239 IU which corresponds to 2.6% of loaded enzyme activity units and indicates a high dynamic binding capacity of Nuvia cPrime resin of ~60 mg mL^−1^. The raw starch digesting ability of some amylases has been ascribed to the binding of starch granules via hydrophobic residues on the surface of amylases^10^. It is theorized that these hydrophobic patches are interacting with the phenolic ring of the functional group of the resin. Elution of amylase with an increased pH and salt concentration showed fractions with a high purity on a SDS PAGE gel (Fig. 3). The eluted enzyme showed an activity of 8800 IU, which represents ~96% yield. Such a high recovery of enzyme is usually only expected from gel permeation or affinity chromatography, but not from other types of chromatographic separations.

The method described is not meant to be replacement for tag technology purification of recombinant proteins, although in some cases it has its advantages. There are many cases where the N or C terminus of a protein is not exposed to solvent and thus the addition of tags is unfeasible. Affinity resins for tagged proteins generally have a low binding capacity (with exception of IMAC resins such as Ni-Sepharose) and are expensive. Mixed-mode resins have a high binding capacity and a comparable price to common ion-exchange resins. We believe that this methodology should be looked at as an important alternative to traditional purification schemes and not just as a replacement for well-known traditional methods. For instance, an interesting use may be found in the purification of recombinant proteins without tags often required for crystallography studies.

## Conclusion

Mixed mode resins are mainly intended for scale-up use and this example may highlight the advantages offered in its use for the purification of raw starch digesting amylases, compared to the classical approach of IEX chromatography followed by polishing step with gel permeation separation. The very high yields and simultaneous concentration and purification may be exploited in the opposite direction as well – the scaling down of the often required purification for testing different mutant variants of enzymes.

## Methods

### Chemicals

All reagents and solvents were purchased from Merck (Darmstadt, Germany) and Sigma-Aldrich (St. Louis, MO, USA) unless otherwise stated. Nuvia cPrime™ resin was purchased from Bio Rad (Hercules, CA, USA).

### Bacterial strains, plasmids and media

The *E. coli* C43 (DE3) strain harbouring pDA-amy plasmid^14^ was used in this work. Frozen stock aliquots containing glycerol prepared from exponential phase cultures grown in Luria-Bertani media (LB) were stored at −80 °C. LB medium, with a composition of 10 g L^−1^ tryptone, 5 g L^−1^ yeast extract and 10 g L^−1^ NaCl, (containing 100 μg mL^−1^ ampicillin) was used for the preinoculum preparation. The compositions of the defined mineral medium, utilising glucose as the sole carbon source, which was used for inoculation and for the bioreactor experiments, as well as the composition of the feed medium for high-cell-density fermentations and the trace elements solution can be found elsewhere^20^.

### Cultivation conditions and analytical procedures

Preinoculum cultures were grown overnight in a 15 mL LB media at 37 °C in a rotary shaker at 250 rpm. To prepare inoculum, 5 mL of preinoculum cultures were transferred aseptically to a 100 mL of defined medium which was incubated at 37 °C for 5 h at 250 rpm. For the bioreactor experiments, 100 mL of inoculum culture was transferred to the bioreactor containing 900 mL of the defined medium. All growth experiments were carried out using a Biostat B bioreactor (Sartorius) equipped with a 2 L fermentation vessel. The end of the batch phase was identified by a reduction in the oxygen consumption rate and an increase in pH. A simple mathematical model based on mass balances and substrate consumption kinetics was used in an open-loop mode to control the specific growth rate at a constant value by an exponential feed medium addition^11^. The exponential feed strategy was continued in order to maintain an almost constant concentration inside the bioreactor and the same specific growth rate. When the DCW reached certain values (15 g L^−1^ and 25 g L^−1^), 0.2 mM IPTG was added as pulse. The glucose feed rate was kept constant at 15 mL h^−1^ after the first pulse, whereas the glucose feed rate was adjusted to 5 mL h^−1^ after the second pulse. The pH was maintained at 7.00 ± 0.05 by adding 15% NH_4_OH solution to the reactor. The temperature was kept at 37 °C and reduced to 25 °C after the induction. The pO_2_ value was maintained at 50% of air saturation by adapting the stirrer speed between 450 and 900 rpm and supplying air (enriched with pure oxygen when necessary) at a space velocity of 2 vvm. The fermentation broth was centrifuged at 10,000 rpm for 20 min at 4 °C using a SL 40 R centrifuge (Thermo Scientific) and the cell-free supernatants were used as a crude enzyme preparation.

Bacterial growth was followed by optical density measurements at 600 nm (OD600). The dry cell weight (DCW) was measured by centrifugation of aliquots of the broth. The pellets were washed twice with deionised water and dried at 110 °C until constant weight.

To quantify the glucose and the recombinant amylase activity during cultures, broth samples were withdrawn, subsequently centrifuged and the supernatant was used. Glucose was analyzed by DNS reagent^21^.

### α-Amylase activity assay and determination of protein concentration

The a-Amylase activity was determined by measuring the formation of reducing sugars released during starch hydrolysis in 50 mM phosphate buffer pH 6.5 and at 75 °C, as described previously^22^. The amount of liberated reducing sugar was determined by the dinitrosalicylic (DNS) acid method^21^. One unit of amylase activity was defined as the amount of enzyme that released 1 μmol of reducing end groups per minute at 75 °C. D-Glucose was used to construct a standard curve.

The protein concentration was determined by the Bradford method^23^ using bovine serum albumin as the protein standard. The abundance of RSDA amongst the rest of extracellular proteins was estimated by analysis of SDS-PAGE gels using ImageJ software (www.rsbweb.nih.gov/ij).

### Purification

The pH of the crude enzyme preparation was adjusted to pH 5.3 and conductivity measurement showed a value of χ ~ 18.3 mS cm^−1^, which corresponds well to conductivity of 50 mM Na-acetate buffer pH 5.3 with 150 mM NaCl. This buffer was used to equilibrate 1 mL column of Nuvia cPrime. 20 mL of extract containing 9200 IU of amylase (66 mg) was loaded on the column. Flow through fractions were collected and tested for activity. The column was washed with 20 ml of starting buffer. Amylase was eluted with 30 mM trisHCl ph 8.0 with 0.5 M NaCl.

### Homology modelling

A homology model was constructed using the SWISS MODEL tool available at the ExPASy server (http://swissmodel.expasy.org/)^24^,^25^.

## Additional Information

**How to cite this article**: Lončar, N. *et al.* Mixed-mode resins: taking shortcut in downstream processing of raw-starch digesting α-amylases. *Sci. Rep.* **5**, 15772; doi: 10.1038/srep15772 (2015).

## Supplementary Material

Supplementary Information

## Figures and Tables

**Figure 1:**
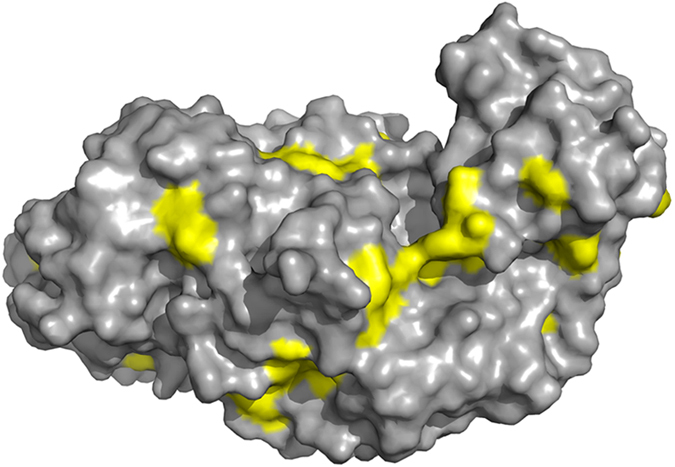
Model structure of *B. licheniformis* 9945a amylase. Exposed tyrosine and tryptophan residues are shown in yellow.
