# Supplementary material for: Mixed-mode resins: taking shortcut in downstream processing of raw-starch digesting α-amylases
Source: Sci Rep. 2015 Oct 23;5:15772. doi: 10.1038/srep15772 (PMC4650330; doi:10.1038/srep15772)
Supplement: Supplementary Information [file srep15772-s1.pdf]

Supplementary Information:

Short Communication,

Mixed-mode resins: taking shortcut in downstream processing of raw-starch digesting α-amylases

Nikola Lončar[*1], Marinela Šokarda Slavić[2], Zoran Vujčić[1,3], Nataša Božić[2]

[1] Department of Biochemistry, Faculty of Chemistry, University of Belgrade, Studentski trg 12-16, Belgrade, Serbia

[2] Centre of Chemistry, Institute of Chemistry, Technology and Metallurgy, University of Belgrade, Studentski trg 12-16, Belgrade, Serbia

[3] Center of Excellence for Molecular Food Sciences, University of Belgrade, Belgrade, Serbia

1. CCD for optimization of purification of amylase directly from fermentation broth

After determining the preliminary range of purification variables according to Bio-Rad's experiments, a central composite design (CCD) was performed with four operation parameters (A-binding pH; B-binding NaCl (mM), C-elution pH, D-elution NaCl (mM). Five replicates at the center point were conducted for calculating the purely experimental uncertainty variance. The optimal levels of these variables were obtained by analyzing the response surface contour plots using the software Design Expert. This facilitated the identification of the following optimal experimental conditions: binding pH 5.3 + 150 mM NaCl and elution pH 8+ 500 mM

[1] Corresponding author: Dr Nikola Lončar
Faculty of Chemistry, University of Belgrade, Studentski trg 12-16, Belgrade, Serbia
e-mail: nloncar@chem.bg.ac.rs
tel: +381 11 3282393

NaCl(Fig. 1). The results of response surface model fitting in the form of ANOVA (analysis of variance) are shown in Table 1. Regression analysis demonstrated that the model was significant, as was evident from the calculated F-value of 25.59 and the probability value (P= 0.0003).

Data from CCD were analyzed with the following second-degree polynomial equation:

$$Yield = -749.73 + 217.16\,A - 0.27\,B + 83.60\,C + 0.04\,D + 0.19\,AB - 4.16\,AC + 0.03\,AD + 0.04\,BC - 0.001\,BD + 0.01\,CD - 23.12\,A^2 - 0.002\,B^2 - 4.06\,C^2 + 0.0001\,D^2$$

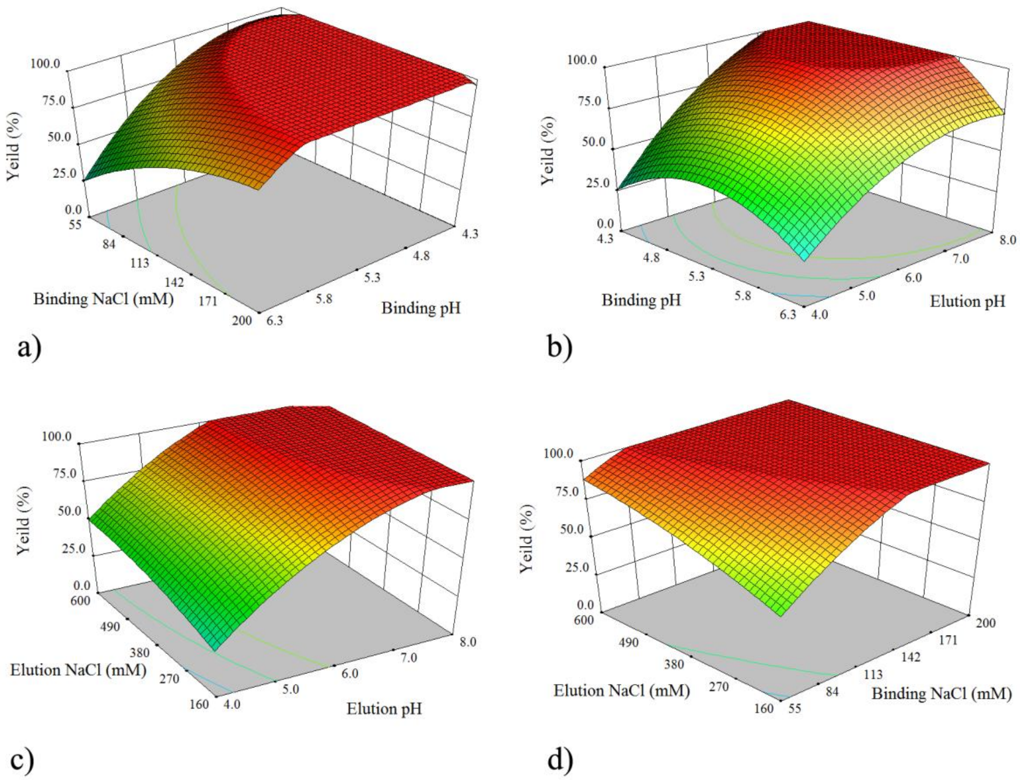

Supplementary Figure S1. Central composite design with 3D response surface plots of the effects of interactions between variables when two variables were set on optimal levels (a) elution pH=8.0, elution NaCl=500 mM, (b) binding NaCl=150 mM, elution NaCl=500mM, (c) binding pH=5.3, binding NaCl=150mM and (d) binding pH=5.3, elution pH=8.0.

Supplementary Table S1. ANOVA for response surface quadratic model of CCD

Source	Sum of Squares	df	Mean Square	F Value	p-value Prob > F	
Model	30720.74	14	2194.34	25.59	0.0003	significant
A-Binding pH	*1512.50*	*1*	*1512.50*	*17.64*	*0.0057*	
B-Binding NaCl	*1012.50*	*1*	*1012.50*	*11.81*	*0.0139*	
C-Elution pH	*13410.34*	*1*	*13410.34*	*156.37*	*< 0.0001*	
D-Elution NaCl	*612.50*	*1*	*612.50*	*7.14*	*0.0369*	
AB	*601.07*	*1*	*601.07*	*7.01*	*0.0382*	
AC	*552.78*	*1*	*552.78*	*6.45*	*0.0441*	
AD	*183.48*	*1*	*183.48*	*2.14*	*0.1939*	
BC	*270.28*	*1*	*270.28*	*3.15*	*0.1262*	
BD	*263.31*	*1*	*263.31*	*3.07*	*0.1303*	
CD	*57.78*	*1*	*57.78*	*0.67*	*0.4431*	
A^2	*7990.87*	*1*	*7990.87*	*93.18*	*< 0.0001*	
B^2	*1961.50*	*1*	*1961.50*	*22.87*	*0.0031*	
C^2	*3936.31*	*1*	*3936.31*	*45.90*	*0.0005*	
D^2	*287.43*	*1*	*287.43*	*3.35*	*0.1169*	
Residual	514.55	6	85.76			
Lack of Fit	*177.75*	*2*	*88.88*	*1.06*	*0.4284*	*not significant*
Pure Error	*336.80*	*4*	*84.20*			
Cor Total	31235.29	20				

2. Supplementary Table S2: Purification of RSDA.

Purification Stage	Total protein (mg)	Total activity (IU)	Specific activity (IU/mg)	Purification (-fold)	Yeild (%)
Fermentation broth	66	9200	139	1	100
Nuvia cPprime chromatography	23	8800	382	2.75	96
